# Crystal structure of 3-(2-hy­droxy­eth­yl)-2-methyl­sulfanyl-6-nitro-3*H*-benzimidazol-1-ium chloride monohydrate

**DOI:** 10.1107/S2056989016013657

**Published:** 2016-08-31

**Authors:** Akoun Abou, Siomenan Coulibali, Rita Kakou-Yao, T. Jérémie Zoueu, A. Jules Tenon

**Affiliations:** a Unité Mixte de Recherche et d’Innovation en Electronique et d’Electricité Appliquées (UMRI EEA), Equipe de Recherche: Instrumentation Image et Spectroscopie (L2IS), DFR–GEE, Institut National Polytechnique Félix Houphouët-Boigny (INP-HB), BP 1093, Yamoussoukro, Côte d’Ivoire, Laboratoire de Cristallographie et Physique Moléculaire, UFR SSMT, Université de Cocody 22 BP 582 Abidjan 22, Côte d’Ivoire; b Laboratoire de Chimie Organique, UFR SSMT, Université de Cocody 22 BP 582 Abidjan 22, Côte d’Ivoire; c Laboratoire de Cristallographie et Physique Moléculaire, UFR SSMT, Université de Cocody 22 BP 582 Abidjan 22, Côte d’Ivoire; d Unité Mixte de Recherche et d’Innovation en Electronique et d’Electricité Appliquées (UMRI EEA)., Equipe de Recherche: Instrumentation Image et Spectroscopie (L2IS), DFR–GEE, Institut National Polytechnique Félix Houphouët-Boigny (INP-HB), BP 1093, Yamoussoukro, Côte d’Ivoire

**Keywords:** crystal structure, benzimidazole derivative, hydrogen bonding, π–π inter­actions

## Abstract

In the cation of the title hydrated molecular salt, C_10_H_12_N_3_O_3_S^+^·Cl^−^·H_2_O, the benzimidazolium ring system is almost planar (r.m.s. deviation = 0.006 Å) and the nitro group is inclined at an angle of 4.86 (9)° to this plane. In the crystal, C—H⋯O hydrogen bonds form centrosymmetric *R*_2_^2^(20) dimers and these are further aggregated through N—H⋯O and O—H⋯Cl hydrogen bonds involving the water mol­ecules and chloride anions. Aromatic π–π stacking inter­actions are also found between two parallel benzene rings or the benzene and imidazolium rings, with centroid–centroid distances of 3.5246 (9) and 3.7756 (9) Å, respectively. Analysis of the bond lengths and comparison with related compounds show that the nitro substituent is not involved in conjugation with the adjacent π-system and hence has no effect on the charge distribution of the heterocyclic ring.

## Abstract 2

The title hydrated salt, C_10_H_12_N_3_O_3_S^+^·Cl^−^·H_2_O, forms centrosymmetric 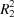
(20) dimers through inter­molecular C—H⋯O hydrogen bonds. These dimers are stacked *via* N—H⋯O and O—H⋯Cl hydrogen bonds involving the water mol­ecules and chloride anions. Offset π–π inter­actions are also present.

## Chemical context   

Numerous compounds with benzimidazole ring systems display versatile pharmacological activities such as anti-viral, anti-helmintic, spasmolitic, anti-hypertensive and vasodilator properties (Akkurt *et al.*, 2006 ▸). Many benzimidazole derivatives also have anti-microbial and anti-fungal activities (Küçükbay *et al.*, 2003 ▸, 2004 ▸; Puratchikody *et al.*, 2008 ▸; Alasmary *et al.*, 2015 ▸). The synthesis of new benzimidazole derivatives is therefore of considerable current inter­est. As part of our studies in this area, the title protonated benzimidazole compound (I) has been synthesized and its mol­ecular structure is presented here.

## Structural commentary   

**Figure 1:**
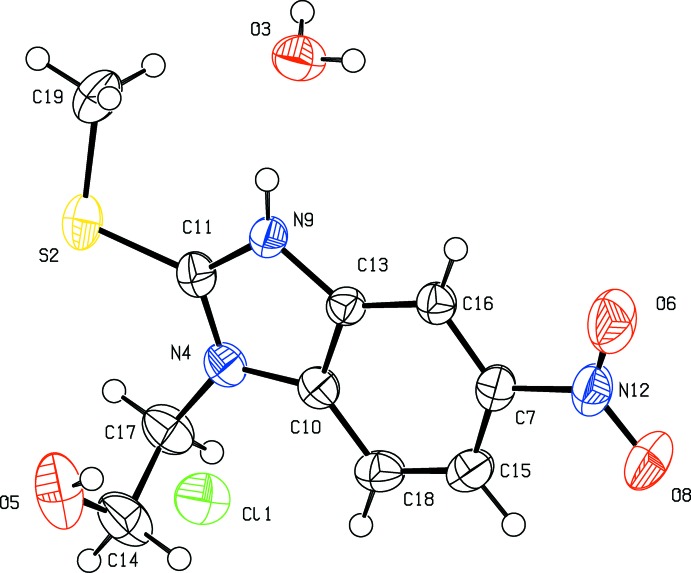
The mol­ecular structure of (I), showing the atomic labelling scheme and displacement ellipsoids drawn at the 50% probability level. H atoms are shown as spheres of arbitrary radius.

The mol­ecular structure of the title compound is shown in Fig. 1 ▸. The nine-membered benzimidazolium ring system (N4/C11/N9/C13/C16/C7/C15/C18/C10) is essentially planar, the maximum deviation from planarity being 0.013 (1) Å for atom N4. In addition, atoms N12, C17 and S2 of the nitro, hy­droxy­ethyl and methyl­sulfanyl substituents lie close to the benzimidazolium ring plane with a maximum deviation of −0.059 (1) Å for atom S2. The least-squares plane of the nitro group (C7/N12/O6/O8) lies close to the benzimidazolium ring system, making a dihedral angle of 4.86 (9)°. In the structure, the bond lengths and angles of the benzimidazolium ring are generally in good agreement with those observed in related structures (Morozov *et al.*, 2004 ▸; Verdan *et al.*, 2009 ▸; Chen *et al.*, 2010 ▸; Yuasa *et al.*, 2010 ▸; Gao *et al.*, 2013 ▸; Samsonov *et al.*, 2013 ▸; Liu *et al.*, 2014 ▸). In addition, the C7—N12 bond length, 1.4667 (19) Å shows that the nitro group is not involved in conjugation with the adjacent π-system and hence has no effect on the charge distribution of the heterocyclic ring. 
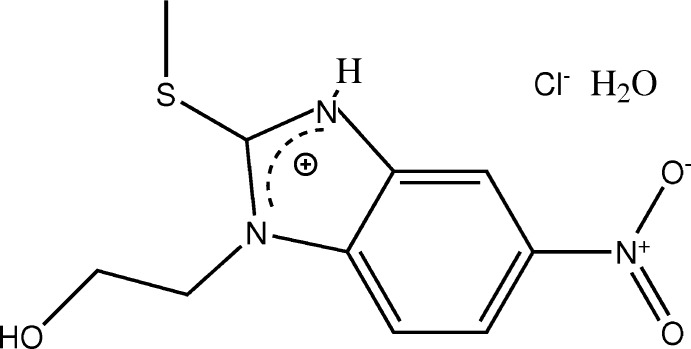


## Supra­molecular features   

**Figure 2:**
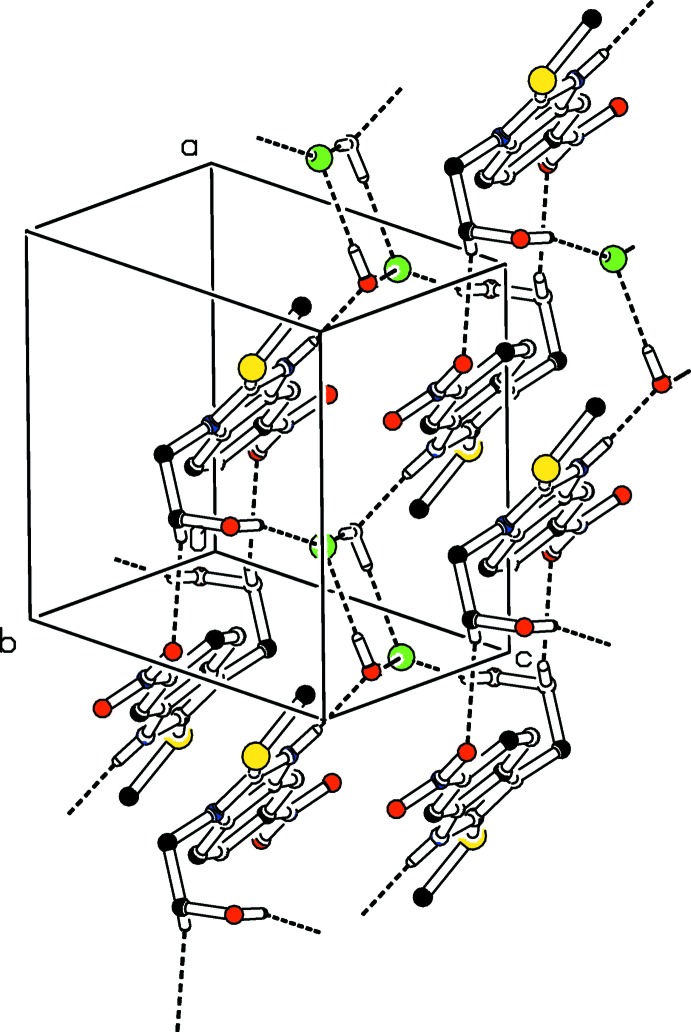
The crystal packing of (I), showing the supra­molecular aggregation resulting from the three-dimensional hydrogen-bonded network. Dashed lines indicate hydrogen bonds. H atoms not involved in hydrogen bonding have been omitted for clarity.

**Figure 3:**
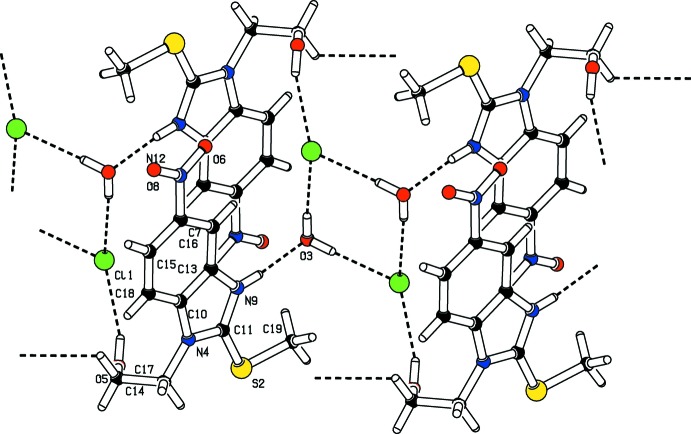
The mol­ecular packing of (I), showing the pattern formed by the water mol­ecules hydrogen bonded to the chloride anions.

**Figure 4:**
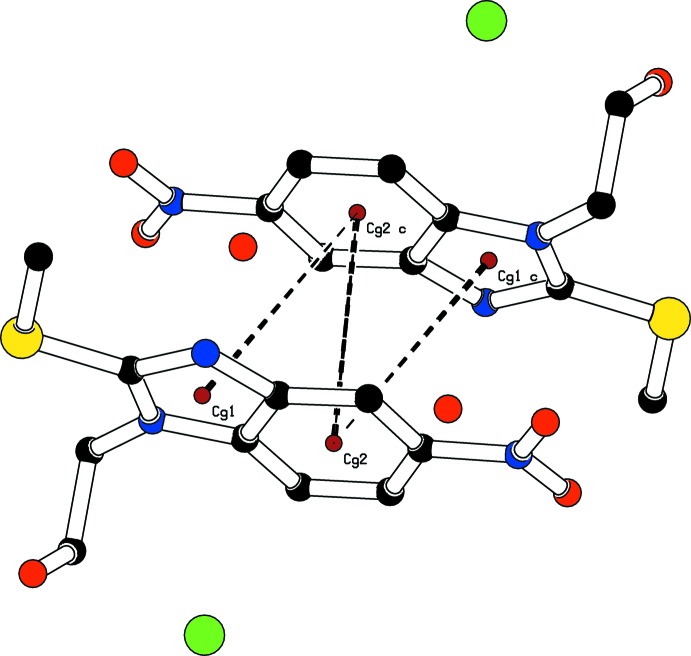
A view of the crystal packing, showing π–π stacking inter­actions (dashed lines). The brown dots are the centroids of the rings. H atoms have been omitted for clarity.

In the crystal, C14—H14*B*⋯O8 hydrogen bonds (Table 1 ▸) link the organic fragments into centrosymmetric dimers with 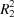
(20) ring motifs along the [100] direction (Fig. 2 ▸). These dimers are further connected along the [100] and [010] directions by N—H⋯O and O—H⋯Cl hydrogen bonds, respectively, generating 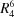
(22) rings. In the latter ring motifs, both the water mol­ecule and the oxygen atom of the hy­droxy­ethyl substituent act as donors with the chloride anion as acceptor. The O3 atom of the water mol­ecule serves as acceptor for the H9 atom of the imidazolium NH group (Fig. 3 ▸). The pattern formed by the water mol­ecules connecting the chloride anions, and forming an 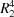
(8) ring, is reminiscent of a parallelogram (Fig. 3 ▸). The supra­molecular aggregation is completed by π–π stacking inter­actions between two parallel benzene rings and between the benzene and imidazolium rings: *Cg*2⋯*Cg*2(1 − *x*, −*y*, −*z*) = 3.5246 (9), *Cg*1⋯*Cg*2(1 − *x*, −*y*, −*z*) = 3.7756 (9) Å, slippage = 1.190 Å *Cg1* and *Cg2* are the centroids of the imidazolium and benzene rings respectively. The centroid–centroid separations are less than 3.8 Å, the maximum regarded as suitable for an effective π–π inter­action (Janiak, 2000 ▸) (Fig. 4 ▸)).

## Database survey   

A CSD search (Web CSD version 5.37; August 19, 2016; Groom *et al.*, 2016 ▸) found eight benzimidazolium structures with substituents at the 1 and 2 positions of the imidazolium ring system (Morozov *et al.*, 2004 ▸; Verdan *et al.*, 2009 ▸; Chen *et al.*, 2010 ▸; Yuasa *et al.*, 2010 ▸; Gao *et al.*, 2013 ▸; Samsonov *et al.*, 2013 ▸; Liu *et al.*, 2014 ▸; Kerimov *et al.*, 2012 ▸). In these structures, the imidazolium rings generally show two long (in the range 1.36–1.40 Å) and two short (1.30–1.34 Å) C—N distances. This pattern is clearly repeated here with N4—C11 = 1.3492 (18) and N9—C11 = 1.3390 (17) Å while N4—C10 = 1.3898 (18) Å and N9—C13 = 1.3867 (16)Å. The sole exception to this pattern is the compound, 2-(4-chloro­phen­yl)-3-[(5-(3,5-di­nitro­phen­yl)-1,3,4-oxa­diazol-2-yl]meth­yl)-1*H*-benzimidazole (Kerimov *et al.*, 2012 ▸), with an imidazolium ring, which reveals three long (1.37–1.39 Å) and one short ( 1.30 Å) C—N bonds, a pattern that is also displayed in benzimidazole structures (Abou *et al.*, 2007 ▸; Yavo *et al.*, 2007 ▸; Kakou-Yao *et al.*, 2007 ▸; Akonan *et al.*, 2010 ▸; Lokaj *et al.*, 2009 ▸).

## Synthesis and crystallization   

2-Chloro­ethanol (1.3 ml, 19.2 mmol) and potassium carbonate (1.32 g, 9.6 mmol) were added to 2-methyl­thio-5-nitro-1*H*-benzimidazole (1.15 g, 4.8 mmol) in dimethyl sulfoxide (DMSO) (10 ml). The reaction mixture was agitated for 5 h at room temperature. 50 ml of water was then added to the reaction mixture, and the products were extracted with di­chloro­methane (3 × 50 ml). The combined organic extracts were washed with ammonium chloride solution (10 g of ammonium chloride in 100 ml of water), dried over Na_2_SO_4_, filtered and evaporated under reduced pressure. The residue was purified by column chromatography on silica gel (elution: methanol/ethyl acetate, 20:80, *v*/*v*). The resulting powder was dissolved in di­chloro­methane and after three days, yellow crystals suitable for single-crystal X-ray diffraction analysis were obtained in 72% yield with a melting point of 425 K.

^1^H NMR (DMSO, 300 MHz) δ(p.p.m.): 2.7 (*s*, 3H, CH_3_); 3 (*s*, 2H, H_2_O); 3.7 (*m*, 2H, CH_2_O); 4.3 (*m*, 2H, CH_2_N); 5 (*t*, 1H, OH); 7.5–8.5 (*m*, 3H, C_6_H_3_).

^13^C (DMSO, 75 MHz) δ (p.p.m.): 114.28 (CH_3_); 47 (CH_2_O); 59 (CH_2_N); 106.56; 110.03; 112.87; 117.13; 136.38; 147.37; 155.52 (C4, C5, C6, C7, C8, C9); 162.23 (C=N).

## Refinement   

Crystal data, data collection and structure refinement details are summarized in Table 2 ▸. The water H atoms were located in a difference Fourier map; their positional parameters and *U*_iso_(H) were refined with O—H distances restrained to be 0.82 Å with a standard deviation of 0.02 Å. Other H atoms were placed in calculated positions [O—H = 0.82, N—H = 0.86, C—H = 0.93 (aromatic), 0.96 (meth­yl) or 0.97 Å (methyl­ene)] and refined using a riding-model approximation with *U*_iso_(H) constrained to 1.2 (amine, aromatic and methyl­ene group) or 1.5 (hydroxyl, methyl group) times *U*_eq_ of the respective parent atom.

## Supplementary Material

Crystal structure: contains datablock(s) I, New_Global_Publ_Block. DOI: 10.1107/S2056989016013657/sj5502sup1.cif

Structure factors: contains datablock(s) I. DOI: 10.1107/S2056989016013657/sj5502Isup2.hkl

Supporting information file. DOI: 10.1107/S2056989016013657/sj5502Isup3.cml

CCDC reference: 1500918

Additional supporting information: crystallographic information; 3D view; checkCIF report

## Figures and Tables

**Table 1:** Hydrogen-bond geometry (Å, °)

*D*—H⋯*A*	*D*—H	H⋯*A*	*D*⋯*A*	*D*—H⋯*A*
O5—H5⋯Cl1	0.82	2.40	3.1840 (15)	161
C17—H17*B*⋯S2	0.97	2.68	3.1514 (18)	110
O3—H3*B*⋯Cl1	0.83 (2)	2.28 (2)	3.1090 (14)	178 (2)
O3—H3*A*⋯Cl1^i^	0.79 (2)	2.37 (2)	3.1561 (14)	174 (2)
C14—H14*B*⋯O8^ii^	0.97	2.60	3.189 (2)	119
N9—H9⋯O3^iii^	0.86	1.85	2.6949 (16)	165

Symmetry codes: (i) 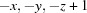; (ii) 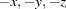; (iii) 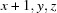.

**Table 2:** Experimental details

Crystal data	
Chemical formula	C_10_H_12_N_3_O_3_S^+^·Cl^−^·H_2_O
*M*_r_	307.75
Crystal system, space group	Monoclinic, *P*2_1_/*c*
Temperature (K)	298
*a*, *b*, *c* (Å)	8.8587 (5), 22.1427 (8), 7.1657 (2)
β (°)	108.497 (3)
*V* (Å^3^)	1332.98 (10)
*Z*	4
Radiation type	Mo *K*α
μ (mm^−1^)	0.46
Crystal size (mm)	0.30 × 0.15 × 0.10

Data collection	
Diffractometer	Nonius KappaCCD
No. of measured, independent and observed [*I* > 2σ(*I*)] reflections	15850, 3856, 3030
*R*_int_	0.029
(sin θ/λ)_max_ (Å^−1^)	0.705

Refinement	
*R*[*F* ^2^ > 2σ(*F* ^2^)], *wR*(*F* ^2^), *S*	0.037, 0.110, 1.06
No. of reflections	3856
No. of parameters	183
No. of restraints	2
H-atom treatment	H atoms treated by a mixture of independent and constrained refinement
Δρ_max_, Δρ_min_ (e Å^−3^)	0.29, −0.24

Computer programs: *COLLECT* (Hooft, 1998 ▸), *DENZO*/*SCALEPACK* (Otwinowski & Minor, 1997 ▸), *SIR94* (Burla *et al.*, 2005 ▸), *PLATON* (Spek, 2009 ▸), *SHELXL2014* (Sheldrick, 2015 ▸), *publCIF* (Westrip, 2010 ▸) and *WinGX* (Farrugia, 2012 ▸).

## supplementary crystallographic information

### Crystal data

**Table:**

C_10_H_12_N_3_O_3_S^+^·Cl^−^·H_2_O	*F*(000) = 640
*M**_r_* = 307.75	*D*_x_ = 1.534 Mg m^−^^3^
Monoclinic, *P*2_1_/*c*	Melting point: 425 K
Hall symbol: -P 2ybc	Mo *K*α radiation, λ = 0.71073 Å
*a* = 8.8587 (5) Å	Cell parameters from 15850 reflections
*b* = 22.1427 (8) Å	θ = 4.1–30.1°
*c* = 7.1657 (2) Å	µ = 0.46 mm^−^^1^
β = 108.497 (3)°	*T* = 298 K
*V* = 1332.98 (10) Å^3^	Block, yellow
*Z* = 4	0.30 × 0.15 × 0.10 mm

### Data collection

**Table:**

Nonius KappaCCD diffractometer	3030 reflections with *I* > 2σ(*I*)
Radiation source: fine-focus sealed tube	*R*_int_ = 0.029
Graphite monochromator	θ_max_ = 30.1°, θ_min_ = 4.1°
f and ω scans	*h* = −12→12
15850 measured reflections	*k* = −31→31
3856 independent reflections	*l* = −9→9

### Refinement

**Table:**

Refinement on *F*^2^	Secondary atom site location: difference Fourier map
Least-squares matrix: full	Hydrogen site location: mixed
*R*[*F*^2^ > 2σ(*F*^2^)] = 0.037	H atoms treated by a mixture of independent and constrained refinement
*wR*(*F*^2^) = 0.110	*w* = 1/[σ^2^(*F*_o_^2^) + (0.0512*P*)^2^ + 0.4116*P*] where *P* = (*F*_o_^2^ + 2*F*_c_^2^)/3
*S* = 1.06	(Δ/σ)_max_ < 0.001
3856 reflections	Δρ_max_ = 0.29 e Å^−^^3^
183 parameters	Δρ_min_ = −0.24 e Å^−^^3^
2 restraints	Extinction correction: SHELXL2014 (Sheldrick 2015, Fc^*^=kFc[1+0.001xFc^2^λ^3^/sin(2θ)]^-1/4^
48 constraints	Extinction coefficient: 0.010 (3)
Primary atom site location: structure-invariant direct methods	

### Special details

**Table:**

Geometry. All esds (except the esd in the dihedral angle between two l.s. planes) are estimated using the full covariance matrix. The cell esds are taken into account individually in the estimation of esds in distances, angles and torsion angles; correlations between esds in cell parameters are only used when they are defined by crystal symmetry. An approximate (isotropic) treatment of cell esds is used for estimating esds involving l.s. planes.
Refinement. Refinement of F^2^ against ALL reflections. The weighted R-factor wR and goodness of fit S are based on F^2^, conventional R-factors R are based on F, with F set to zero for negative F^2^. The threshold expression of F^2^ > 2σ(F^2^) is used only for calculating R-factors(gt) etc. and is not relevant to the choice of reflections for refinement. R-factors based on F^2^ are statistically about twice as large as those based on F, and R- factors based on ALL data will be even larger.

### Fractional atomic coordinates and isotropic or equivalent isotropic displacement parameters (Å^2^)

**Table:**

	*x*	*y*	*z*	*U*_iso_*/*U*_eq_	
Cl1	0.13738 (5)	0.08560 (2)	0.42268 (6)	0.05068 (14)	
S2	0.57871 (5)	0.21143 (2)	0.26622 (7)	0.04747 (14)	
O3	−0.15176 (14)	0.05681 (6)	0.56505 (18)	0.0472 (3)	
N4	0.36705 (14)	0.12789 (5)	0.06943 (17)	0.0345 (3)	
O5	0.1477 (2)	0.20574 (6)	0.1828 (3)	0.0718 (4)	
H5	0.1668	0.1773	0.2603	0.108*	
O6	0.45778 (19)	−0.14042 (6)	0.3012 (2)	0.0634 (4)	
C7	0.34801 (17)	−0.05387 (6)	0.1321 (2)	0.0342 (3)	
O8	0.24519 (18)	−0.14988 (6)	0.0528 (2)	0.0679 (4)	
N9	0.56755 (14)	0.08753 (5)	0.29958 (17)	0.0314 (2)	
H9	0.6563	0.0838	0.3931	0.038*	
C10	0.33709 (16)	0.06625 (6)	0.06760 (19)	0.0313 (3)	
C11	0.50505 (17)	0.13941 (6)	0.2131 (2)	0.0333 (3)	
N12	0.35085 (17)	−0.11931 (6)	0.1646 (2)	0.0430 (3)	
C13	0.46514 (15)	0.04077 (6)	0.21301 (19)	0.0288 (3)	
C14	0.1141 (2)	0.18374 (9)	−0.0101 (3)	0.0561 (5)	
H14A	0.0477	0.2127	−0.1014	0.067*	
H14B	0.0548	0.1463	−0.0229	0.067*	
C15	0.21856 (18)	−0.02957 (7)	−0.0142 (2)	0.0395 (3)	
H15	0.1372	−0.0546	−0.0881	0.047*	
C16	0.47534 (16)	−0.02074 (6)	0.2500 (2)	0.0310 (3)	
H16	0.5606	−0.0383	0.3459	0.037*	
C17	0.26321 (19)	0.17270 (8)	−0.0631 (2)	0.0443 (4)	
H17A	0.2347	0.1583	−0.1977	0.053*	
H17B	0.3207	0.2104	−0.0550	0.053*	
C18	0.21161 (17)	0.03179 (7)	−0.0491 (2)	0.0390 (3)	
H18	0.1269	0.0493	−0.1461	0.047*	
C19	0.7761 (2)	0.19682 (8)	0.4278 (3)	0.0560 (5)	
H19A	0.7703	0.1756	0.5421	0.084*	
H19B	0.8311	0.2344	0.4673	0.084*	
H19C	0.8327	0.1727	0.3606	0.084*	
H3B	−0.076 (2)	0.0647 (10)	0.524 (3)	0.060 (6)*	
H3A	−0.152 (3)	0.0212 (7)	0.573 (4)	0.070 (8)*	

### Atomic displacement parameters (Å^2^)

**Table:**

	*U*^11^	*U*^22^	*U*^33^	*U*^12^	*U*^13^	*U*^23^
Cl1	0.0492 (2)	0.0521 (3)	0.0514 (2)	−0.00027 (17)	0.01683 (18)	0.00853 (17)
S2	0.0580 (3)	0.02568 (18)	0.0586 (3)	−0.00229 (15)	0.0185 (2)	−0.00067 (15)
O3	0.0382 (6)	0.0495 (7)	0.0520 (7)	0.0023 (5)	0.0116 (5)	0.0069 (5)
N4	0.0362 (6)	0.0320 (6)	0.0354 (6)	0.0051 (4)	0.0116 (5)	0.0048 (4)
O5	0.1054 (13)	0.0430 (7)	0.0891 (11)	0.0073 (7)	0.0620 (10)	0.0007 (7)
O6	0.0788 (10)	0.0336 (6)	0.0715 (9)	−0.0019 (6)	0.0152 (7)	0.0062 (6)
C7	0.0393 (7)	0.0307 (6)	0.0376 (7)	−0.0053 (5)	0.0193 (6)	−0.0049 (5)
O8	0.0661 (9)	0.0436 (7)	0.0917 (11)	−0.0218 (6)	0.0217 (8)	−0.0198 (7)
N9	0.0322 (6)	0.0264 (5)	0.0335 (6)	−0.0009 (4)	0.0075 (4)	−0.0016 (4)
C10	0.0325 (6)	0.0329 (6)	0.0299 (6)	0.0013 (5)	0.0119 (5)	0.0003 (5)
C11	0.0380 (7)	0.0283 (6)	0.0360 (7)	0.0018 (5)	0.0150 (5)	0.0011 (5)
N12	0.0507 (8)	0.0323 (6)	0.0540 (8)	−0.0091 (5)	0.0278 (6)	−0.0085 (6)
C13	0.0276 (6)	0.0301 (6)	0.0296 (6)	−0.0013 (5)	0.0103 (5)	−0.0019 (5)
C14	0.0517 (10)	0.0490 (10)	0.0725 (12)	0.0175 (8)	0.0269 (9)	0.0167 (9)
C15	0.0352 (7)	0.0463 (8)	0.0379 (7)	−0.0097 (6)	0.0129 (6)	−0.0094 (6)
C16	0.0330 (6)	0.0297 (6)	0.0322 (6)	−0.0002 (5)	0.0128 (5)	−0.0003 (5)
C17	0.0451 (8)	0.0437 (8)	0.0447 (8)	0.0122 (7)	0.0151 (7)	0.0151 (7)
C18	0.0317 (7)	0.0491 (8)	0.0335 (7)	0.0004 (6)	0.0066 (5)	−0.0011 (6)
C19	0.0531 (10)	0.0394 (8)	0.0723 (12)	−0.0149 (7)	0.0153 (9)	−0.0057 (8)

### Geometric parameters (Å, º)

**Table:**

S2—C11	1.7194 (14)	N9—H9	0.8600
S2—C19	1.794 (2)	C10—C18	1.388 (2)
O3—H3B	0.833 (16)	C10—C13	1.3932 (18)
O3—H3A	0.792 (16)	C13—C16	1.3849 (18)
N4—C11	1.3492 (18)	C14—C17	1.506 (2)
N4—C10	1.3898 (18)	C14—H14A	0.9700
N4—C17	1.4758 (18)	C14—H14B	0.9700
O5—C14	1.405 (3)	C15—C18	1.379 (2)
O5—H5	0.8200	C15—H15	0.9300
O6—N12	1.219 (2)	C16—H16	0.9300
C7—C16	1.3853 (19)	C17—H17A	0.9700
C7—C15	1.393 (2)	C17—H17B	0.9700
C7—N12	1.4667 (19)	C18—H18	0.9300
O8—N12	1.2251 (18)	C19—H19A	0.9600
N9—C11	1.3390 (17)	C19—H19B	0.9600
N9—C13	1.3867 (16)	C19—H19C	0.9600
			
C11—S2—C19	101.51 (8)	C17—C14—H14A	109.2
H3B—O3—H3A	105 (2)	O5—C14—H14B	109.2
C11—N4—C10	108.48 (11)	C17—C14—H14B	109.2
C11—N4—C17	126.35 (13)	H14A—C14—H14B	107.9
C10—N4—C17	125.16 (12)	C18—C15—C7	119.69 (13)
C14—O5—H5	109.5	C18—C15—H15	120.2
C16—C7—C15	124.82 (13)	C7—C15—H15	120.2
C16—C7—N12	117.17 (13)	C13—C16—C7	114.40 (12)
C15—C7—N12	118.01 (13)	C13—C16—H16	122.8
C11—N9—C13	108.53 (11)	C7—C16—H16	122.8
C11—N9—H9	125.7	N4—C17—C14	111.36 (13)
C13—N9—H9	125.7	N4—C17—H17A	109.4
C18—C10—N4	131.27 (13)	C14—C17—H17A	109.4
C18—C10—C13	122.29 (13)	N4—C17—H17B	109.4
N4—C10—C13	106.44 (11)	C14—C17—H17B	109.4
N9—C11—N4	109.42 (12)	H17A—C17—H17B	108.0
N9—C11—S2	128.39 (11)	C15—C18—C10	116.82 (13)
N4—C11—S2	122.18 (11)	C15—C18—H18	121.6
O6—N12—O8	123.47 (15)	C10—C18—H18	121.6
O6—N12—C7	118.44 (13)	S2—C19—H19A	109.5
O8—N12—C7	118.09 (15)	S2—C19—H19B	109.5
C16—C13—N9	130.92 (12)	H19A—C19—H19B	109.5
C16—C13—C10	121.98 (12)	S2—C19—H19C	109.5
N9—C13—C10	107.10 (11)	H19A—C19—H19C	109.5
O5—C14—C17	112.04 (17)	H19B—C19—H19C	109.5
O5—C14—H14A	109.2		
			
C11—N4—C10—C18	179.32 (14)	C11—N9—C13—C10	0.53 (15)
C17—N4—C10—C18	−0.1 (2)	C18—C10—C13—C16	0.1 (2)
C11—N4—C10—C13	−1.53 (14)	N4—C10—C13—C16	−179.10 (12)
C17—N4—C10—C13	179.07 (13)	C18—C10—C13—N9	179.86 (12)
C13—N9—C11—N4	−1.51 (15)	N4—C10—C13—N9	0.61 (14)
C13—N9—C11—S2	178.15 (11)	C16—C7—C15—C18	−0.1 (2)
C10—N4—C11—N9	1.90 (15)	N12—C7—C15—C18	179.95 (13)
C17—N4—C11—N9	−178.70 (13)	N9—C13—C16—C7	179.86 (13)
C10—N4—C11—S2	−177.78 (10)	C10—C13—C16—C7	−0.51 (19)
C17—N4—C11—S2	1.6 (2)	C15—C7—C16—C13	0.5 (2)
C19—S2—C11—N9	11.50 (16)	N12—C7—C16—C13	−179.58 (12)
C19—S2—C11—N4	−168.88 (13)	C11—N4—C17—C14	−106.73 (18)
C16—C7—N12—O6	5.1 (2)	C10—N4—C17—C14	72.56 (19)
C15—C7—N12—O6	−174.94 (15)	O5—C14—C17—N4	60.0 (2)
C16—C7—N12—O8	−174.90 (14)	C7—C15—C18—C10	−0.2 (2)
C15—C7—N12—O8	5.0 (2)	N4—C10—C18—C15	179.29 (14)
C11—N9—C13—C16	−179.79 (14)	C13—C10—C18—C15	0.3 (2)

### Hydrogen-bond geometry (Å, º)

**Table:**

*D*—H···*A*	*D*—H	H···*A*	*D*···*A*	*D*—H···*A*
O5—H5···Cl1	0.82	2.40	3.1840 (15)	161
C17—H17*B*···S2	0.97	2.68	3.1514 (18)	110
O3—H3*B*···Cl1	0.83 (2)	2.28 (2)	3.1090 (14)	178 (2)
O3—H3*A*···Cl1^i^	0.79 (2)	2.37 (2)	3.1561 (14)	174 (2)
C14—H14*B*···O8^ii^	0.97	2.60	3.189 (2)	119
N9—H9···O3^iii^	0.86	1.85	2.6949 (16)	165

Symmetry codes: (i) −*x*, −*y*, −*z*+1; (ii) −*x*, −*y*, −*z*; (iii) *x*+1, *y*, *z*.
